# Differential Expression Patterns of SLCO Solute Carriers in Human Breast Cancer Cell Lines and Tumour Samples

**DOI:** 10.1111/jcmm.70838

**Published:** 2025-09-03

**Authors:** Rachel Telfer‐Sutherland, Louise Page, Annette Meeson, Simon Lowes

**Affiliations:** ^1^ Biosciences Institute Newcastle University, International Centre for Life, Central Parkway Newcastle UK; ^2^ Translational and Clinical Research Institute Newcastle University Newcastle UK; ^3^ Northern Centre for Breast Research, Breast Screening and Assessment Unit, Queen Elizabeth Hospital Gateshead Health NHS Foundation Trust Gateshead UK

**Keywords:** breast cancer, chemotherapeutic drugs, drug transport, oestrogen transport, SLCO, solute transporter superfamily

## Abstract

The cellular uptake of nutrients essential for cell growth and survival is facilitated by solute carrier (SLC) transporters. Members of the SLCO subfamily of SLCs mediate the uptake of substrates relevant to breast cancer (BC), including steroid hormones and anticancer drugs. Accumulating evidence suggests that altered expression of these transporters may affect BC pathogenesis by influencing cell proliferation and anticancer drug resistance. In this study, we investigated differential expression of 11 SLCO transporters using semi‐quantitative and quantitative PCR in MCF‐7 and MDA‐MB‐231 BC cell lines and in human BC tissue samples. Eight SLCO transporters were expressed in at least one cell line. Of these, SLCO1B1 and SLCO1B3 showed higher expression in MDA‐MB‐231 than MCF‐7 cells. Conversely, SLCO2A1, SLCO4C1 and SLCO5A1 showed higher expression in MCF‐7 than MDA‐MB‐231 cells. Quantitative PCR analysis of 18 patients' BC tissue samples revealed variable expression of a number of SLCO transporters, regardless of patients having been treated with or without endocrine therapy prior to tumour excision. Proliferation and gene expression studies were conducted on the cell lines following exposure to β‐estradiol, which stimulated cell proliferation in MCF‐7 cells, as well as causing a significant increase in SLCO4C1 gene expression.

**Abbreviations**
ABC: ATP‐binding cassette
BC: breast cancer
ER: oestrogen receptor
HER2: human epidermal growth factor receptor 2
MDR: multidrug resistance
OATP: organic anion transporting peptide
PR: progesterone receptor
SLC: solute carrier family of uptake transporters

## 1 Introduction

Mechanisms that underpin the pathogenesis of breast cancer (BC) are highly complex. Gaining insights into this is further complicated by the biological heterogeneity between breast tumours, a factor that is recognised to contribute to treatment failure [1]. One mechanism that may contribute directly to disease pathogenesis and the effectiveness of therapy is the balance between uptake and efflux transporters, which, in coordination, facilitate the bi‐directional transport of solutes into and out of cells. ABC transport proteins such as ABCB1, ABCG2 and ABCC1 mediate the efflux of chemotherapeutic agents out of cells and are established as important mediators of BC progression and treatment [2], including contributing to multidrug resistance (MDR) [3, 4, 5, 6].

Although there is now a reasonable level of understanding of the role of efflux transporters in BC cells, comparatively little is known about the expression and role of transporters mediating uptake of substrates into cells, the so‐called solute carrier (SLC) transporters. The SLC superfamily is the second largest group of membrane proteins in humans, comprising over 400 proteins arranged into 65 subfamilies [7]. Physiologically, they contribute to homeostasis by facilitating the intracellular uptake of a wide variety of endogenous and exogenous anionic, cationic and zwitterionic substrates into cells using electrochemical and concentration gradients [7, 8, 9, 10]. The range of substrates for SLC transporters is diverse, and many members within the same family share overlapping substrate specificity [9, 11].

The SLCO subfamily, which incorporates the transporters also known as organic anion transporting peptides (OATP), is of particular interest in BC, as many of its members transport substrates important for BC progression and treatment, including steroid hormone metabolites and anticancer drugs [8, 9, 12, 13, 14, 15, 16]. There are 11 human OATPs, which are classified into 6 families, and further into subfamilies, based on their amino acid sequence similarities [8]. The OATPs all share the same basic protein structure, comprising 12 cell membrane‐spanning helices, with N‐ and C‐terminal bundles located on the intracellular side, and the substrate binding pocket within the C‐bundle [17].

The fact that many OATPs transport steroid metabolites is of potential significance in BC, since the majority of BCs are hormone responsive [18], whereby intracellular sex steroids, principally oestrogens, promote tumour proliferation. Hormone responsiveness of BCs is dependent upon oestrogen receptor (ER) and/or progesterone receptor (PR) expression within the tumours. ER and PR expression is routinely tested on diagnostic tissue samples using immunohistochemistry, with around 83% of tumours ER positive, and 65% PR positive [19]. Hormone receptor status, together with expression levels of human epidermal growth factor receptor 2 (HER2), helps guide treatment decisions and prognosis. ‘Triple negative’ tumours, which lack expression of all three receptors (ER‐, PR‐ and HER2‐), have a poorer prognosis [20]. Patients who have triple negative tumours, or HER positive tumours (irrespective of ER/PR status), often require chemotherapy [21]. Studies in different tissues have established that oestrogens are substrates for at least 5 of the 11 human transporters (SLCO1A2/OATP1A2, SLCO1B1/OATP1B1, SLCO1B3/OATP1B3, SLCO2B1/OATP2B1 and SLCO4C1/OATP4C1), which, depending on their expression in BC cells, could implicate their potential involvement in BC development and treatment [8, 9, 12, 15, 16, 22, 23, 24].

Although the expression of a few select SLC transporters in various BC cell lines and tissues has been demonstrated, the available evidence is piecemeal. This study aimed to provide a comprehensive overview of SLCO expression in hormone‐responsive (MCF‐7) and hormone‐non‐responsive (MDA‐MB‐231) BC cell lines and human BC tissue samples to gain an indication of differential expression patterns. Expression levels of select SLCOs known to transport oestrogens were also measured following exposure of BC cells to β‐estradiol to gain some insight into the potential for inducibility of transporter expression.

## 2 Methods

### 2.1 Human Tissue Samples and Ethical Approval

All research involving patients was performed according to the tenets of the Declaration of Helsinki. Fresh breast tumour samples were obtained at the Breast Screening and Assessment Unit, Gateshead Health NHS Foundation Trust, United Kingdom, from patients with biopsy‐proven BC. The samples were obtained from two cohorts, all of whom gave their written consent for their tissue to be used in this project. The first cohort was patients undergoing ultrasound‐guided vacuum‐assisted excision (VAE) of their tumours as part of a separate clinical research study (PICASSO; REC number 18/NW/0168). VAE is a procedure carried out under local anaesthesia using a 7‐ or 10‐gauge needle with vacuum‐assisted suction permitting minimally invasive lesion excision; the aim of the PICASSO study was to investigate the feasibility of VAE in patients who were deemed clinically unsuitable for surgical excision of their primary tumour based on their co‐morbidities. These were patients who would ordinarily have been treated with endocrine therapy as primary disease control. All patients had therefore already received primary endocrine therapy prior to tumour excision by VAE (Table 1). The provision of breast tissue samples for further research was built into the PICASSO study protocol, so at the time of the VAE, some of the freshly excised tumour samples were obtained for use in this study. The remainder of the tissue was sent to the pathology team as part of standard care. The second cohort from whom tissue samples were obtained was patients undergoing mastectomy for primary treatment of their BC; following surgery, the mastectomy specimens were transferred to the breast unit, where ultrasound‐guided core biopsies of the tumour were taken by a breast radiologist (SL). The mastectomy specimen was then sent to the pathology team as part of standard care. Ethical approval for obtaining these samples was covered under an overarching agreement between Gateshead Health NHS Foundation Trust and Newcastle Biobank (REC number 17/NE/0361). All tissue samples were placed directly into RNA‐later from a total of 18 patients aged 44 to 85 years, including one male patient. Basic demographic information and tumour characteristics are summarised in Table 1.

**TABLE 1:** Patient demographics and tumour characteristics.

Patient	Date collected	Excision type	Histology	Grade	Gender	Age	ER	HER2	Prior treatment	Treatment Duration
Patient 1	17/12/18	VAE	IDC	1	F	81	+	−	Letrozole	3 months
Patient 2	12/11/19	VAE	IDC	1	F	78	+	−	Letrozole	2 months
Patient 3	16/12/19	VAE	IDC	3	F	80	+	−	Letrozole	66 months
Patient 4	23/12/19	VAE	IDC^a^	2	F	82	+	−	Letrozole then tamoxifen	76 months
Patient 5	02/11/20	VAE	IDC	2	F	58	+	−	Letrozole	8 months
Patient 6	19/11/20	Mastectomy	IDC	3	F	80	+	−	None	N/A
Patient 7	23/11/20	Mastectomy	IDC	2	M	72	+	−	None	N/A
Patient 8	14/11/20	Mastectomy	IDC	1	F	63	+	−	None	N/A
Patient 9	21/01/21	Mastectomy	IDC	2	F	63	+	−	None	N/A
Patient 10	25/01/21	VAE	IDC	2	F	64	+	−	Letrozole	5 weeks
Patient 11	26/01/21	Mastectomy	IDC	3	F	64	+	−	None	N/A
Patient 12	10/02/21	Mastectomy left	IDC	2	F	82	+	−	None	N/A
	10/02/21	Mastectomy right	IDC	2	F	82	+	+	None	N/A
Patient 13	16/02/21	Mastectomy	ILC	2	F	50	+	−	None	N/A
Patient 14	18/02/21	Mastectomy	IDC	3	F	75	−	+	None	N/A
Patient 15	02/03/21	Mastectomy	IDC	1	F	44	+	−	None	N/A
Patient 16	04/03/21	Mastectomy	IDC	2	F	85	+	−	None	N/A
Patient 17	04/03/21	Mastectomy	IDC	2	F	73	+	−	None	N/A
Patient 18	03/06/21	Mastectomy	IDC	2	F	61	+	+	None	N/A

*Note:* Grey highlighting denotes samples where the patient had received endocrine therapy. Patients 1 to 5 and 10 underwent VAE; the remainder had mastectomy. Patient 12 had bilateral mastectomies.

Abbreviations: ER, oestrogen receptor; F, female; HER2, human epidermal growth factor receptor 2; IDC, invasive ductal carcinoma; ILC, invasive lobular carcinoma; M, male.

^a^ Mixed IDC and mucinous carcinoma.

### 2.2 Cell Lines and Culture

BC cell lines used were MCF‐7 (purchased from ECACC, Salisbury, UK) and MDA‐MB‐231 (provided by MD Anderson, Houston, Texas, USA). Both cell lines were validated via STR profiling using PowerPlex 16 HS PCR Amplification Kit (Promega, Wisconsin, USA) according to the manufacturer's instructions. The MCF‐7 and MDA‐MB‐231 cells were cultured as described previously [25].

### 2.3 RNA Isolation

Total RNA was isolated from cell lines using the RNeasy Micro Kit (Qiagen, Manchester, UK) and from patient tumour samples using TRI Reagent (Merck, USA) followed by the RNeasy Micro Kit (Qiagen, Manchester, UK). All protocols were performed as per manufacturers' instructions.

### 2.4 Positive Controls

Human total kidney RNA was purchased from ThermoFisher Scientific (Massachusetts, USA). Human brain RNA was isolated from tissue from the Parkinson's UK Brain Bank, UK, and kindly gifted by Dr. Ilse Pienaar, Sussex University, UK. Human placental tissue was provided by the Newcastle Uteroplacental Tissue Bank (REC 16/NE/0167), Newcastle University, UK. Human heart tissue was provided by the Newcastle Institute of Transplantation Tissue Biobank, UK, ethics agreement number IOT028. The human liver cancer cell line, HepG2, was kindly gifted by Professor Matthew Wright, Newcastle University, UK. The human lung squamous cell carcinoma cell line, NCI‐H520, was kindly gifted by Professor Alastair Greystoke, Newcastle University, UK. Human testis RNA was purchased from Amsbio (Abingdon, UK). RNA was isolated from placental and heart tissue using TRI Reagent (Merck, USA). RNA was isolated from HepG2 and NCI‐H520 cells using the RNeasy Mini Kit (Qiagen, Manchester, UK). Details of positive controls used for each transporter, selected based on publications describing the expression of relevant SLCO transporters in these tissue types, are provided in Table S1.

### 2.5 cDNA Synthesis

cDNA was synthesised using the Bioline cDNA Synthesis Kit (Scientific Laboratory Supplies, Nottingham, UK) for cell lines and patient‐derived tumour samples as per the manufacturer's instructions.

### 2.6 Semi‐Quantitative Polymerase Chain Reaction (Sq‐PCR)

Sq‐PCR was performed as described previously [26]. Details of primer sequences and reaction conditions are listed in Table S2. All PCR reactions included GAPDH loading controls and positive/non‐template negative controls.

### 2.7 Quantitative Real‐Time Polymerase Chain Reaction (qPCR)

qPCR experiments were performed as described previously [27]. The reaction was set up at 95°C for 10 min followed by 40 cycles of 95°C for 15 s and 60°C for 1 min. Relative mRNA expression was calculated using the comparative ΔΔ*C*_t_ method. TaqMan probes were purchased from Applied Biosystems (California, USA). Assay IDs for each target are listed in Table S3.

### 2.8 Primer Design

Primers were designed using the National Centre for Biotechnology Information (NCBI) Primer‐BLAST website search tool. This allowed the generation of primer sequences specific to the gene of interest from the NCBI refseq accession numbers. Primers were designed using the following criteria where possible: a primer length of 18 to 22 bp, allowing specificity and assisting with binding at the annealing temperature; an optimum *T*_m_ (melting temperature) of 52°C to 58°C to prevent secondary annealing at higher temperatures; a GC content of 40% to 60%, including the presence of a GC clamp, to aid in specific binding; product lengths did not exceed 200 bp.

### 2.9 Primer Validation

DNA Sequencing was used to determine the specificity of the PCR primers for the gene of interest. Following the PCR reaction, DNA was extracted from gel fragments using a QIAquick Gel Extraction Kit (Qiagen, Germany) according to the manufacturer's protocol. The purified DNA sample was sent to Source BioScience (UK) for sequencing. The resulting sequence data were compared with transporter gene sequences available on the NCBI database.

### 2.10 Oestrogen Exposure and MTS Assay

Cells were plated into 96‐well plates at 500 cells/well in 200 μL/well complete media containing 10% charcoal stripped FBS (ThermoFisher Scientific, Massachusetts, USA) and incubated for 24 h. The media was then removed and replaced with media containing 10% charcoal stripped FBS and 1 nM β‐estradiol (Sigma‐Aldrich, Missouri, USA). Controls included untreated cells and a media only control. Cells were incubated at 37°C with 5% CO_2_ for 6 days. Following incubation, media was removed, and an MTS assay was performed as previously described to assess cell proliferation [27].

### 2.11 SLCO4C1/OATP4C1 Immunostaining

1 × 10^6^ cells were fixed in 5 mL methanol for 15 min, followed by permeabilisation in 5 mL 0.1% Triton‐X‐100 for 5 min. Immunofluorescence staining was performed as previously described [28]. An SLCO4C1/OATP4C1 Antibody (SLCO4C1 Rabbit IgG Polyclonal Antibody, ThermoFisher Scientific, USA; subsequently discontinued) was used at a 1:50 dilution and the Fluorescein (FITC)‐conjugated Goat Anti‐Rabbit IgG secondary antibody AB_2337972 (Jackson ImmunoResearch Europe Ltd., Ely, UK) was used at a 1:25 dilution. The epitope for the SLCO4C1 antibody is 516–544 amino acids from the C‐terminus region, which is intracellular. Controls included no primary antibody and unstained cells. Cell suspensions were analysed by BD LSRFortessa cell analyser and BD FACS Diva software (BD Biosciences, New Jersey, USA).

### 2.12 Microscopy Imaging

5 × 10^3^ cells were seeded in each well of 8‐well chamber slides (BD Falcon) in complete Dulbecco's Modified Eagle Medium (DMEM) and incubated at 37°C with 5% CO_2_ for 48 h. Cells were fixed with 200 μL per well of methanol or 4% paraformaldehyde (PFA). Cells were permeabilised by the addition of 0.1% Triton‐X‐100 and blocked in 5% normal goat serum in 1× phosphate‐buffered saline (PBS) for 1 h at room temperature. Antibody was added at the correct concentration (1:50, 1:75, 1:100, 1:150, 1:200, 1:250, 1:350) diluted in 1× PBS containing 1% normal goat serum and incubated overnight. The cells were washed three times in 1× PBS for 5 min before being incubated in a 1:25 dilution of FITC‐conjugated goat anti‐rabbit secondary antibody in the dark for 1 h. Cells were washed before being mounted using VECTASHIELD anti‐fade mounting medium (Vector Laboratories, USA). Slides were analysed using a Zeiss AxioImager (Carl Zeiss AG, Germany).

### 2.13 Statistical and Data Analysis

Statistical analysis was performed using IBM SPSS Statistics 24 (Chicago, Illinois, USA) and graphs were generated on GraphPad Prism version 8 (GraphPad Software, San Diego, USA). All experiments were performed in triplicate for cell lines pertaining to three different cell passages. Results are presented as mean ± SD. All statistical analyses were performed on Log_2_ transformed data. Statistical significance was determined by one‐sample *t*‐test, independent samples *t*‐test, or paired‐samples *t*‐test as appropriate. All results are reported as significant at *p* < 0.05.

## 3 Results

### 3.1 Sq‐PCR Detection of SLCO Transporter Expression in MCF‐7 and MDA‐MB‐231 Cell Lines and Human BC Tissue

**FIGURE 1:**
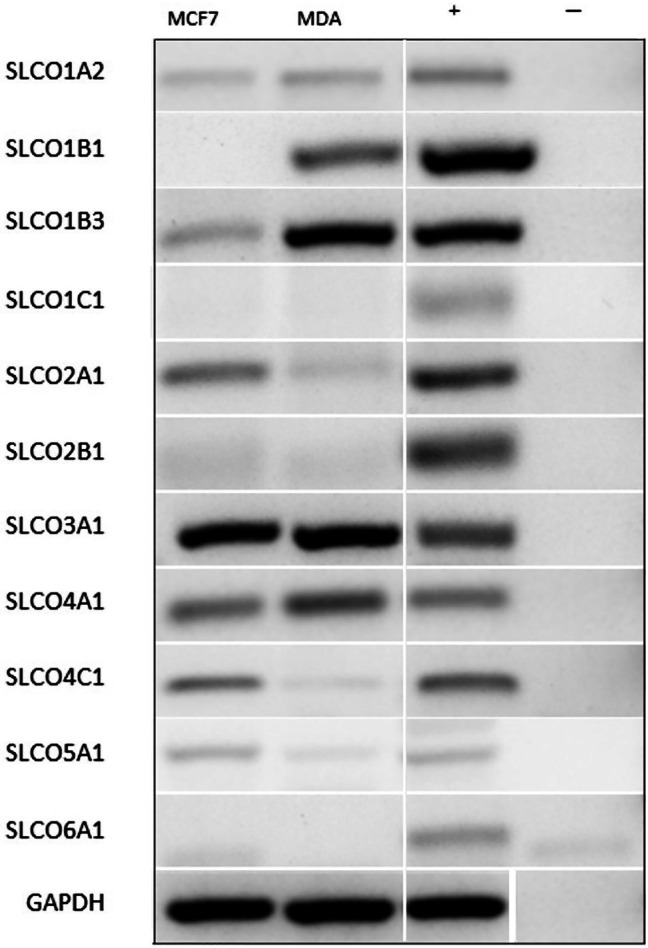
sq‐PCR analysis of 11 SLCO transporter expression in MCF7 and MDA‐MB‐231 (labelled as MDA) cell lines and the housekeeping gene GAPDH. (See Table S1 for positive controls (+) and Table S2 for product sizes). (−) negative control. White bars separating the bands indicate where the original lane position was re‐ordered for image presentation.

Differential mRNA expression of the 11 human SLCO genes was initially compared between the MCF‐7 and MDA‐MB‐231 cells using sq‐PCR (Figure 1). Three of the genes, SLCO1C1/OATP1C1, SLCO2B1/OATP2B1 and SLCO6A1/OATP6A1, showed no/very low expression in either cell line. SLCO1B1/OATP1B1 showed high expression in MDA‐MB‐231 cells but was not appreciably detected in MCF‐7 cells. SLCO1B3/OATP1B3 was expressed in both cell lines but was higher in MDA‐MB‐231. SLCO4C1/OATP4C1, SLCO5A1/OATP5A1 and SLCO2A1/OATP2A1 were also expressed in both cell lines but higher in MCF‐7 than MDA‐MB‐231. The remaining transporters, SLCO1A2/OATP1A2, SLCO4A1/OATP4A1 and SLCO3A1/OATP3A1, showed similar expression in both cell lines (Figure 1).

**FIGURE 2:**
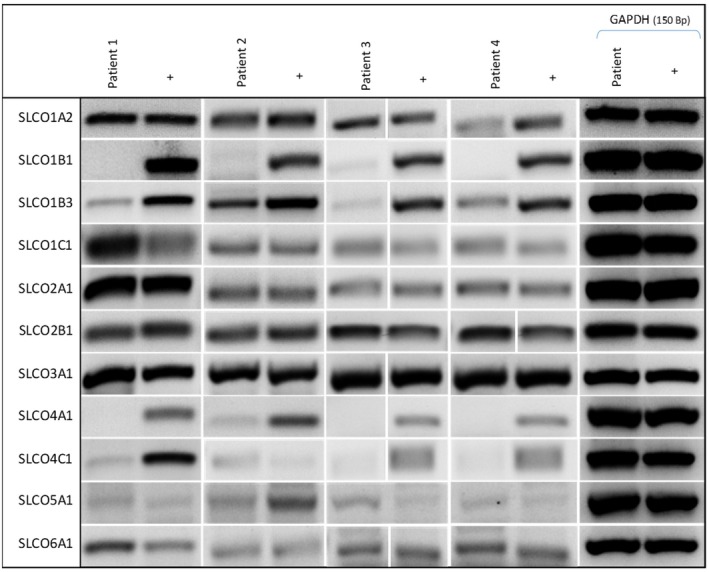
Semi‐quantitative PCR showing expression of SLCO transporter genes in four human breast cancer tissue samples from patients undergoing VAE of their tumours. (+) positive control (GAPDH) was used as a loading control for all experiment. A representative example of GAPDH expression in included in the figure. A non‐template control was use in all PCR reactions (see Table S1 for positive controls and Table S2 for product sizes). White bars separating the bands indicate where the original lane position was re‐ordered for image presentation.

All 11 SLCO transporters were then investigated using sq‐PCR in samples from the first four patients, all of whom were part of the first cohort of tissue samples who had undergone therapeutic VAE of their tumours. As these were all patients deemed unfit for general anaesthetic and open surgical excision, all had received endocrine therapy for between 2 and 76 months prior to excision (Table 1 and Figure 2). Across all four patients, the overall expression pattern of the SLCO subtypes was similar. SLCO1A2/OATP1A2, SLCO1C1/OATP1C1, SLCO2A1/OATP2A1, SLCO2B1/OATP2B1 and SLCO3A1/OATP3A1 were consistently expressed in all patients, with patient 1 showing a greater band intensity of both SLCO1C1/OATP1C1 and SLCO2A1/OATP2A1. SLCO1B3/OATP1B3 was detected in all four samples, but showed apparent variability in detection levels between patients, with the highest levels seen in patient 2. There was no or negligible detection of SLCO1B1/OATP1B1, SLCO4A1/OATP4A1, SLCO4C1/OATP4C1. All tumours expressed SLCO5A1/OATP5A1, but expression was weak and variable across patients; the positive control was heart cDNA, as despite testing several positive controls, only this showed any detectable expression, in agreement with previous findings [29]. Despite no demonstrable expression in the cell lines, SLCO6A1/OATP6A1 was detectable in tissue samples but showed variable expression (Figure 2).

### 3.2 Q‐PCR Detection of SLCO Transporter Expression in MCF‐7 and MDA‐MB‐231 Cell Lines and Human BC Tissue

Expression of the 11 SLCO transporters was then investigated using qPCR in both cell lines and in the human BC tissue samples. Tissue samples were used from all 18 patients from both cohorts: cohort 1 (VAE; patients 1–5 and patient 10) and cohort 2 (mastectomy; patients 6–9 and 11–18) (Table 1). From 18 patients there were a total a 19 tissue samples, since patient 12 underwent bilateral mastectomy. Because of limited tissue availability, preference was given to SLCOs deemed to be of most interest based on their expression in the cell lines (Figure 1) and in the preliminary sq‐PCR findings in the first 4 patient samples (cohort 1; Figure 2), as well as their likely clinical relevance in relation to their known substrates. Thus SLCO1A2, SLCO1B1, SLCO1B3, SLCO1C1, SLCO2A1, SLCO2B1, SLCO3A1 and SLCO4C1 were assessed in all tissue samples, whereas it was possible to analyse only 12 tumour samples for SLCO4A1 and SLCO5A1, and only 4 samples for SLCO6A1.

**FIGURE 3:**
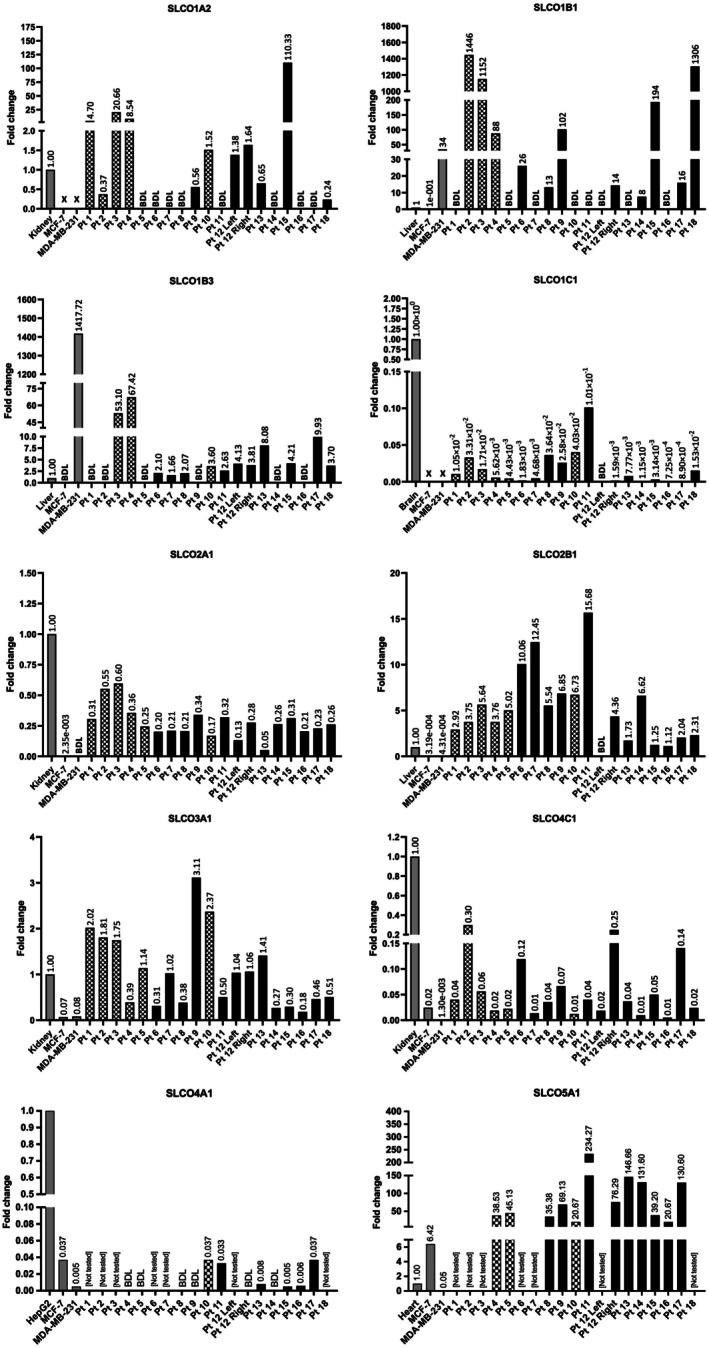
Relative mRNA expression of SLCO Transporters in MCF‐7 and MDA‐MB‐231 cell lines and human breast cancer tissue using qPCR. BDL, below detection limits; Pt, patient. Numbers provided above vertical bars = fold change in mRNA expression. All expression values are normalised to the housekeeping gene β‐Actin and are expressed as fold change relative to a reference sample (kidney, liver, brain or heart cDNA). Checked bars denote samples when patients had received endocrine therapy prior to excision (all had VAE) and black bars denote samples where patient had not (all had mastectomy). Due to small sample size, further statistical analysis was not possible.

The data analysis is presented in Figure 3 for 10 of the 11 genes; because of the small number of samples for SLCO6A1, the findings are described but not shown in figure form.

#### 3.2.1 Cell Line qPCR

Of the 11 genes, 3 were below the detection limit for both cell lines but were detectable in the positive control: SLCO1A2/OATP1A2, SLCO1C1/OATP1C1 (Figure 3), and SLCO6A1 (not shown). Positive controls, also referred to as calibrator samples, are given in Table S1. Both SLCO1B1/OATP1B1 and SLCO1B3/OATP1B3 showed considerably higher expression in MDA‐MB‐231 cells compared to the calibrator sample (34‐fold (*p* = 0.013) and 1418‐fold (*p* = 0.001) higher, respectively), while in MCF‐7 cells, SLCO1B1/OATP1B1 expression was detectable but lower than that of the calibrator (*p* = 0.423), and SLCO1B3/OATP1B3 was below the detection limit (Figure 3). SLCO5A1 showed 6.4‐fold higher expression in the MCF7 compared to the calibrator samples (*p* = 0.003), whereas in the MDA‐MB‐231 expression was detectable but lower than the calibrator (*p* = 0.6037), and there was a significant difference in the expression between the two cell lines (*p* = 0.0013). Moreover, comparison of the fold change of the two cell lines relative to the calibrator suggests that SLCO2A1/OATP2A1 was expressed at a higher level in the MCF‐7 than the MDA‐MB‐231 cells (*p* value for SLCO2A1 not stated as MDA‐MB‐231 values fall below the detection limit). Conversely, no significant difference in the expression of SLCO4A1/OATP4A1 (*p* = 0.078), SLCO4C1/OATP4C1 (*p* = 0.222), SLCO2B1/OATP2B1 (*p* = 0.831) or SLCO3A1/OATP3A1 (*p* = 0.661) was detected between the cell lines (Figure 3). Low SLCO2B1 expression was detected in both cell lines using qPCR where it had not been identified by sq‐PCR.

#### 3.2.2 BC Tissue Sample qPCR

Despite being below the detection limits in both cell lines, SLCO1A2 expression was identified in 11 of 19 samples, which varied from 0.24‐fold expression (Pt 18) to 110.33‐fold increased expression (Pt 15) relative to the reference sample (kidney). In the remaining 8 tumours, expression was below the detection limit. 11 tumours showed expression of SLCO1B1, of between 8‐ to 1446‐fold higher than the reference sample (liver), with the remaining 8 samples below the detection limit. Of note, patient 12, who had bilateral mastectomies for IDC tumours of differing hormone receptor status (left breast: ER+ HER2− and right breast: ER + HER2+), showed expression of SLCO1B1 in the right (ER + HER2+) and not the left (ER + HER2‐) tumour. Expression of SLCO1B1 was below the detection limit in 8 patient tumours (Pt 1, 5, 7, 10, 11, 12 [left], 13 and 16). In 13 tumours, SLCO1B3 expression was consistently above that of the reference sample (liver) (ranging from 1.66‐ to 67.42‐fold higher). No expression was detected in 6 patient tumours (Pt: 1, 2, 5, 9, 14 and 17). SLCO1C1 was expressed at a very low level in all tumours (ranging from 7.25 × 10^−4^ to 1.0 × 10^−1^) compared to the reference sample (brain), except for Pt 12 [left] where expression was below the detection limit. SLCO2A1 was expressed at a consistently low level in all 19 tumours, ranging from 0.05 to 0.6‐fold of the reference sample (kidney). Higher expression of SLCO2B1 was displayed by 18 of 19 tumour samples compared to the reference sample (liver), ranging from 1.12–15.68‐fold higher, but expression was below the detection limit for Pt 12 [left]. Expression of SLCO3A1 was expressed by all 19 tumours, however, this expression was variable. In 9 samples the expression was lower than that of the reference sample (kidney) (0.17–0.51‐fold) and in the remaining 10 samples, expression was higher (1.02 to 3.11‐fold relative to the reference). SLCO4C1 was also expressed at low level within all 19 tumours, ranging between 0.01‐ and 0.3‐fold of the reference sample (kidney). SLCO4A1 expression was detected in only 6 of the 12 samples assessed, and in all cases well below that of the positive control (highest fold change 0.037). SLCO5A1 was expressed in all 12 samples tested, with variable levels, though in all cases higher than that seen in the reference sample (heart) (ranging from 20.67 to 234.27). Consistent with the findings in the two cell lines, SLCO6A1 was undetectable in the four samples tested, which comprised tumours from two patients who had undergone endocrine therapy prior to tumour excision, and two therapy‐naïve patients (data not shown).

Overall there were no identifiable common patterns of individual SLCO gene expression related to specific tumour types, such as oestrogen receptor status (though all tumours were ER+ except patient 14), HER2 status (all HER2‐ except patients 12 right, 14, and 18), histological subtype (though all were IDC except patient 13, which was ILC), or treatment with endocrine therapy.

### 3.3 Effect of β‐Estradiol Exposure on MCF‐7 and MDA‐MB‐231 Cell Proliferation and SLCO Transporter Expression

Because the expression of oestrogen‐transporting SLCO genes had been confirmed in both cell lines, and because MCF‐7 cells are regarded as a hormone‐responsive cell line, it was next investigated whether exposure to an oestrogen can stimulate cell growth, and if so whether there is a differential effect between MCF‐7 and MDA‐MB‐231 cells. First, the effect on MCF‐7 and MDA‐MB‐231 cells following exposure to β‐estradiol was investigated using an MTS proliferation assay. A range of β‐estradiol concentrations (1–100 nM) were titrated to determine the optimal concentration required to promote cell proliferation (data not shown). Cells were exposed to a final concentration of 1 nM β‐estradiol for 6 days as previously described [30, 31].

**FIGURE 4:**
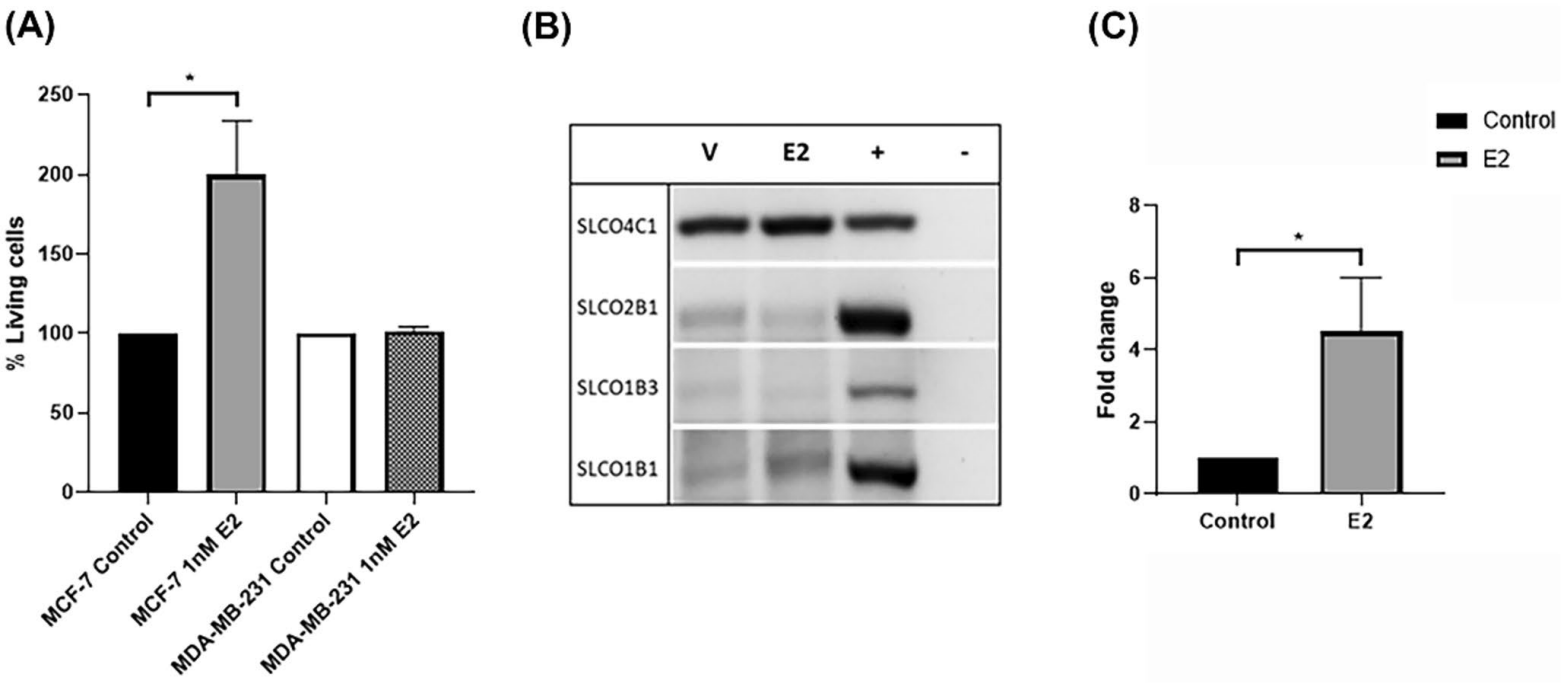
Impact of oestrogen stimulation on SLCO transporter expression and cell proliferation. (A) Percentage of living MCF‐7 and MDA‐MB‐231 cells following stimulation with 1 nM β‐estradiol measured via MTS assay. The figure is representative of four independent experiments and four cell passages. Values are presented as mean ± SEM. Statistical significance was calculated using a one‐sample or independent samples *t*‐test (*p < 0.05). (B) Semi‐quantitative PCR results show expression of SLCO4C1, SLCO2B1, SLCO1B3 and SLCO1B1 in MCF‐7 cells following 6 days' treatment with 1 nM β‐estradiol (E2) or the vehicle only control (V). (+) positive control (GAPDH) was used as a loading control for all experiments. A non‐template control was used in all PCR reactions (−). (C) Quantitative PCR showing relative mRNA expression of SLCO4C1 in MCF‐7 cells following stimulation for 6 days with 1 nM β‐estradiol (E2). Data are represented as mean ± SEM of five independent experiments. Statistical significance was calculated using a one‐sample *t*‐test (*p < 0.05). SLCO2B1, SLCO1B3, and SLCO1B1 levels were all negligible or below detection limits in both non‐stimulated and stimulated cells (data not shown).

In MCF‐7 cells, 1 nM β‐estradiol induced a significant increase in cell proliferation above that of the vehicle‐only control (*p* = 0.036), whereas in MDA‐MB‐231, cell proliferation remained at a similar level to that of the vehicle‐only control (*p* = 0.855) (Figure 4A).

Because exposure to β‐estradiol had resulted in an increase in MCF‐7 cell proliferation, it was next investigated whether β‐estradiol exposure also played a role in the expression of certain oestrogen‐transporting SLCOs. Those established as transporters of oestrogen conjugates are SLCO1A2/OATP1A2, SLCO1B1/OATP1B1, SLCO1B3/OATP1B3, SLCO2B1/OATP2B1, and SLCO4C1/OATP4C1 [15]. Of these, all except SLCO1A2/OATP1A2 showed some degree of expression in the cell lines, therefore SLCO4C1/OATP4C1, SLCO2B1/OATP2B1, SLCO1B3/OATP1B3, and SLCO1B1/OATP1B1 were selected for further experiments. Expression was measured following 6 days' exposure to 1 nM β‐estradiol. Using sq‐PCR, none of the transporters showed a significant change in expression relative to controls (Figure 4B). qPCR was then carried out to investigate this further. Although it confirmed no change in expression of SLCO1B1/OATP1B1, SLCO1B3/OATP1B3, or SLCO2B1/OATP2B1, whose levels were all negligible or below detection limits in the both non‐stimulated and stimulated cells (data not shown), β‐estradiol exposure did result in a significant increase in the expression of SLCO4C1 (*p* = 0.015) (Figure 4C). Further experiments therefore focused on SLCO4C1/OATP4C1.

### 3.4 Analysis of SLCO4C1/OATP4C1 Protein Expression via Immunostaining

**FIGURE 5:**
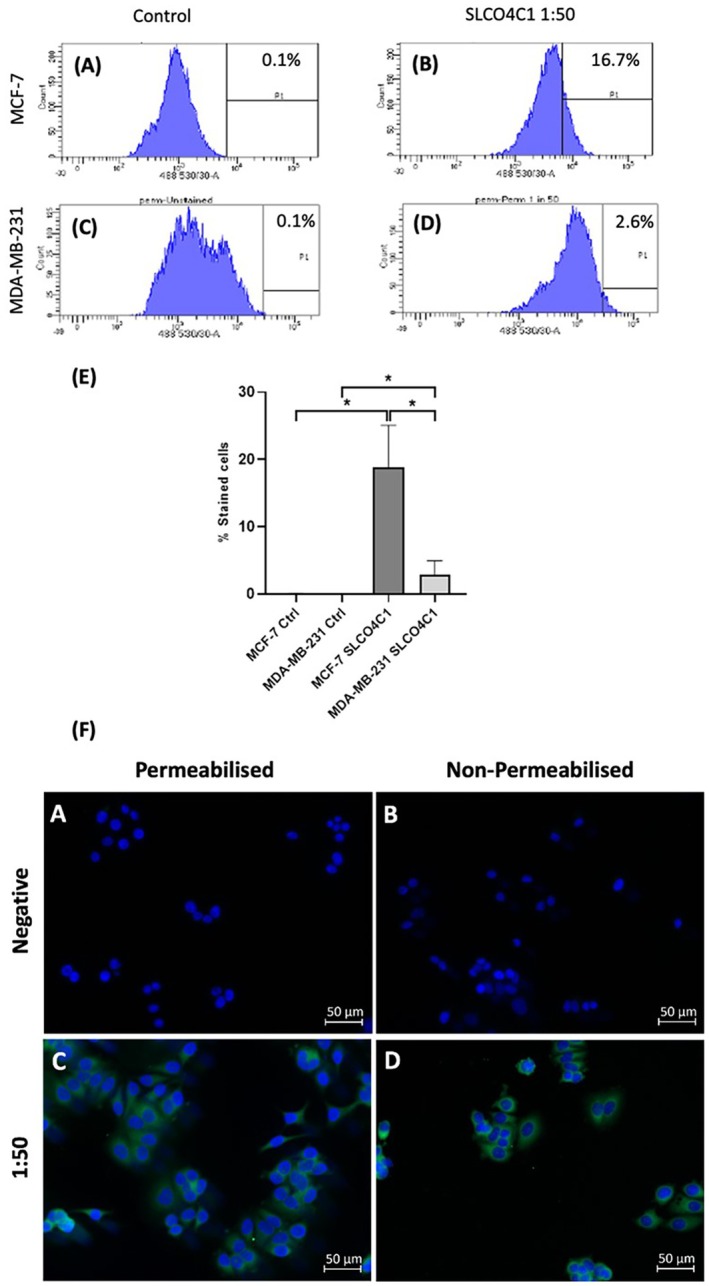
Analysis of protein expression of SLCO4C1 reveals differential expression of protein between MDA‐MB‐231 and MCF7, with expression being higher in MCF7. Representative FACS data plots of methanol‐fixed Triton‐X‐100 permeabilised MCF‐7 and MDA‐MB‐231 cells (A–D). (A) MCF‐7 and (C) MDA‐MB‐231 controls immunostaining without primary antibody. (B) MCF‐7 and (D) MDA‐MB‐231 cells stained with 1:50 dilution of SLCO4C1 antibody. (E) Graphical representation of FACS staining data *n* = 3. Immunocytochemistry images showing SLCO4C1 expression in MCF‐7 cells (F). Results of immunostaining of MCF7 cells in culture (F); staining at a 1:50 dilution in MCF‐7 cells following methanol fixation and permeabilisation with Triton‐X‐100 (F, panel C) and non‐permeabilisation (F panel D). Unstained controls are shown in (F panels A and B). Scale bars = 50 μm.

Based on the sq‐PCR and qPCR analysis, and on the results of the β‐estradiol stimulation, SLCO4C1/OATP4C1 expression was investigated further in both cell lines using immunostaining. Representative FACS plots are shown in Figure 5A–D. MCF‐7 cells showed significantly higher mean expression of SLCO4C1/OATP4C1 than in MDA‐MB‐231 cells, with 18.8% versus 2.9% of cells staining (*p* = 0.036; *n* = 3) (Figure 5E). MCF‐7 cells, both permeabilised (Figure 5F) and non‐permeabilised (Figure 5F), were also stained in culture to determine localisation of expression of SLCO4C1. Expression of SLCO4C1 was detected in MCF‐7 cells under both conditions, with no appreciable difference between the two, and appeared to be localised to the cell membrane.

## 4 Discussion

The findings from the this study, together with two previous smaller studies investigating a narrower range of SLCOs [18, 32], now allow an overview of the expression patterns in MCF‐7 and MDA‐MB‐231 cells to be mapped out to assess key commonalities and differences that might reflect their different phenotypes, on the basis that MCF‐7 cells are regarded as a model for hormone‐responsive BC, whereas MDA‐MB‐231 cells are regarded as a model for hormone‐non‐response BC. One might therefore expect that oestrogen‐transporting SLCOs would demonstrate higher expression in MCF‐7 cells than MDA‐MB‐231, but this was not always the case. With both SLCO1B1/OATP1B1 and SLCO1B3/OATP1B3, for example, not only are these both consistently highly expressed in MDA‐MB‐231 cells, with little/no expression in MCF‐7 cells, the levels in MDA‐MB‐231 cells are much higher than in the positive control, liver. This is noteworthy, because these transporters were until recently thought to have been liver‐specific, and it is in liver where they are regarded as having their greatest functional significance, as important mediators of drug disposition [33]. The marked differential expression between MDA‐MB‐231 and MCF‐7 cells is a similar finding to that of Banerjee et al. [18], who also found that SLCO1B1/OATP1B1 and SLCO1B3/OATP1B3 were both highly expressed in MDA‐MB‐231 cells, with little/no expression in MCF‐7.

Similarly, in the human BC tissue samples, the expression of both SLCO1B1/OATP1B1 and SLCO1B3/OATP1B3 was variable. SLCO1B1/OATP1B1 showed very high expression in of the tissue samples, in all cases much higher than the positive control (liver), yet was below the detection limit in the remainder. SLCO1B3/OATP1B3 expression levels were in all cases lower than in MDA‐MB‐231, but it was expressed in most of the tissue samples. For both genes, there were no discernible patterns of expression levels in relation to ER status of the tumours or previous/concurrent endocrine therapy. Despite this variation in expression in BC tissue, and the apparent paradoxical overexpression in MDA‐MB‐231 cells, in vitro work in BC cell lines has indicated that SLCO1B3/OATP1B3 has a role in cellular function. Tang et al. [33] found that knockdown of OATP1B3 in MDA‐MB‐231 cells was associated with a significant increase in cell proliferation, migration, and invasion. Overexpression of OATP1B3 in the BC cell line BT549, on the other hand, was associated with a significant reduction in cell proliferation, migration and invasion. Furthermore, in BC patients, those with high levels of tumour SLCO1B3/OATP1B3 expression were found to be more likely to be lymph node‐negative, have early (stage I) disease, and have histologically low grade (Grade 1) tumours [34]. This supports an earlier study, in which immunohistochemical staining for OATP1B3 was carried out on IDCs [35], where positive SLCO1B3/OATP1B3 immunoreactivity of tumours (defined as > 10% of cells staining positively) was inversely correlated with tumour size and was significantly associated with decreased risk of recurrence. This overall suggests that SLCO1B3/OATP1B3 expression is beneficial for disease prognosis, but the mechanism underpinning this potential protective effect is unclear. SLCO1B3/OATP1B3, as well as SLCO1B1/OATP1B1, also transports a range of clinically relevant chemotherapeutic agents [16, 36, 37, 38, 39], so could play a role in facilitating or limiting drug uptake into some tumours.

Some of the genes tested, namely SLCO2A1/OATP2A1, SLCO2B1/OATP2B1, SLCO3A1/OATP3A1, SLCO4C1/OATP4C1 and SLCO5A1/OATP5A1, were generally found to be consistently expressed across most or all tumour samples, suggesting these may play a more consistent functional role within BC cells, if indeed they are functionally expressed. Again, however, there was little correlation of expression in the BC tissue with the cell lines, with negligible or no expression of SLCO2A1/OATP2A1, SLCO2B1/OATP2B1, SLCO3A1/OATP3A1 and SLCO4C1/OATP4C1 in either cell line and some expression of SLCO5A1/OATP5A1 in MCF‐7 cells but negligible levels in MDA‐MB‐231 cells. What is already known about these transporters from other studies may give us additional clues as to their likely importance in BC. SLCO2A1/OATP2A1 is regarded as ubiquitously expressed and is predominantly recognised as a prostaglandin transporter [40], suggesting it may not be directly important in BC; SLCO2B1/OATP2B1 transports estrone sulphate and the precursor dehydroepiandrosterone sulphate (DHEAS) [41], making it a potential candidate in hormone‐dependent BC pathogenesis. It also transports a wide range of drugs, though none directly relevant to BC treatment [42]. SLCO3A1/OATP3A1 also transports estrone sulphate [41], and there is further evidence to suggest it plays a functional role in BC cells; in a clinical study using peripheral blood samples from elderly patients with ER‐positive BC treated with aromatase inhibitors, it was found that several different single nucleotide polymorphisms (SNPs) of SLCO3A1 were associated with different responses to aromatase inhibitor treatment, some resulting in better treatment outcomes, and some associated with worse outcomes [43]. SLCO4C1/OATP4C1 is also a transporter of estrone sulphate [44], and although the data in this study show it is expressed at relatively low levels in the tumour samples, similar to the baseline levels in MCF‐7 cells, it was shown to be inducible in the MCF‐7 s, with significantly increased expression and significantly increased cellular proliferation following treatment with β‐estradiol (metabolically active oestrogen). Immunostaining for SLCO4C1 confirmed in MCF‐7 localisation at the cell membrane as would be expected for an membrane transporter; although no other studies have demonstrated SCLO4C1 localisation in breast tissue, studies in human and rat kidney also show it is localised at the cell membrane [45, 46]. The inducibility of SLCO4C1 in response to β‐estradiol suggests that this transporter, and potentially other SLCOs, have modifiable expression, which might well influence their function in vivo.

Despite SLCO5A1/OATP5A1 being expressed at consistently high levels in all the tested tumour samples relative to the cell lines and the positive control, its endogenous function remains unclear, with no clinically relevant substrates identified [41], though a previous study has also detected it in BC cells [47].

The remaining transporter genes, SLCO1C1/OATP1C1, SLCO4A1/OATP4A1 and SLCO6A1/OATP6A1, showed no or low/negligible expression in most of the BC tissue samples and the cell lines, suggesting these are unlikely to play a functionally significant role in BC. This is reinforced by their known substrate specificities, which are not directly linked to BC pathogenesis; SLCO1C1/OATP1C1 transports thyroid hormones, SLCO4A1/OATP4A1 transports thyroid hormones, prostaglandins, and bile salts and SLCO6A1/OATP6A1 is currently poorly characterised [41].

The differential genes expression patterns across the different human tissue samples almost certainly reflects the known heterogeneity of BC, but at the same time, the apparent lack of consistency of expression in any tumour types hinders our understanding of the significance of the contribution of individual SLCOs to individual patients' disease. This variability of expression is not unique to our dataset, however [18, 32]. This heterogeneity may also reflect the difficulty in treating BC as a single disease entity; whereas, most tumours are treated with primary surgical excision, some are treated using chemotherapy in the neoadjuvant, adjuvant or palliative settings, where response to treatment may vary between individuals in ways that are not predictable based on the standard clinical tumour markers [48].

One other consideration regarding apparent differential expression patterns based on PCR is the fact that different polymorphic variants may exist. Multiple SNPs have been identified OATP1B1, OATP1B3, OATP2B1 and OATP1A2, for example, which often result in different transporter functions and could have pharmacokinetic implications for the many drugs they transport [49]. Because these all transport steroid hormones, there could also be potential implications for BC pathogenesis. This is reinforced by the study mentioned above, where SLCO3A1 SNPs were associated with different responses to aromatase inhibitor treatment [43]. An SLCO1B1 SNP has also been associated with increased circulating oestrogen levels in women with BC receiving aromatase inhibitor therapy [50]. Another study has shown that a further SLCO1B1 SNP is associated with increased plasma docetaxel concentrations in women with BC, thought to be a result of reduced systemic tissue uptake of the drug [51]. Although these studies demonstrate the systemic impact on SLCO SNPs through serum sampling, there is a paucity of information on the potential impact of SNPs at a BC tissue level. Further work to characterise the full extent of SNPs at both a systemic and tissue level may help to increase our understanding of the clinical significance of these transporters in BC pathogenesis and treatment. Although in this study, primer design was based on the common published isoforms, this does open raise the possibility of differential specificity depending on variants in different patient samples. Of note a cancer‐specific variant of SLCO1B3 has been detected in colon, pancreatic and ovarian cancers [52, 53], with one study reporting it is also present in BC [54].

One challenge in pursuing a more comprehensive analysis of SLCO variants is access to fresh human tissue, not only in terms of the number and heterogeneity of different tumour samples but also the volume of tissue available from each tumour for performing multiple analyses. Nevertheless, a larger number of tumour samples and the ability to test for multiple polymorphic variants may offer a more complete mapping of transporter profiles, allowing certain profile ‘signatures’ particular to certain tumour subtypes to be established, which might 1 day help to serve as a prognostic tool.

## Author Contributions

**Rachel Telfer‐Sutherland:** formal analysis (equal), investigation (equal), methodology (equal), writing – original draft (equal), writing – review and editing (equal). **Louise Page:** formal analysis (equal), investigation (equal), writing – review and editing (equal). **Annette Meeson:** conceptualization (equal), formal analysis (equal), funding acquisition (supporting), investigation (equal), methodology (lead), supervision (equal), writing – original draft (equal), writing – review and editing (equal). **Simon Lowes:** conceptualization (equal), formal analysis (equal), funding acquisition (lead), methodology (equal), supervision (equal), writing – original draft (equal), writing – review and editing (equal).

## Consent

All patients gave their written consent for samples of their tissue to be used in this project.

## Conflicts of Interest

The authors declare no conflicts of interest.

## Supporting information


**Tables S1–S3:** jcmm70838‐sup‐0001‐TableS1‐S3.docx.

## Data Availability

The datasets used and/or analysed during this study are available from the corresponding author on reasonable request.

## References

[1] D. Hanahan and R. A. Weinberg , “Hallmarks of Cancer: The Next Generation,” Cell 144, no. 5 (2011): 646–674. 21376230 10.1016/j.cell.2011.02.013

[2] F. Leonessa and R. Clarke , “ATP Binding Cassette Transporters and Drug Resistance in Breast Cancer,” Endocrine‐Related Cancer 10, no. 1 (2003): 43–73. 12653670 10.1677/erc.0.0100043

[3] L. A. Doyle , W. Yang , L. V. Abruzzo , et al., “A Multidrug Resistance Transporter From Human MCF‐7 Breast Cancer Cells,” Proceedings of the National Academy of Sciences of the United States of America 95, no. 26 (1998): 15665–15670. 9861027 10.1073/pnas.95.26.15665 PMC28101

[4] E. L. Volk , K. M. Farley , Y. Wu , F. Li , R. W. Robey , and E. Schneider , “Overexpression of Wild‐Type Breast Cancer Resistance Protein Mediates Methotrexate Resistance,” Cancer Research 62, no. 17 (2002): 5035–5040. 12208758

[5] J. R. Riordan , K. Deuchars , N. Kartner , N. Alon , J. Trent , and V. Ling , “Amplification of P‐Glycoprotein Genes in Multidrug‐Resistant Mammalian Cell Lines,” Nature 316, no. 6031 (1985): 817–819. 2863759 10.1038/316817a0

[6] M. Munoz , M. Henderson , M. Haber , and M. Norris , “Role of the MRP1/ABCC1 Multidrug Transporter Protein in Cancer,” IUBMB Life 59, no. 12 (2007): 752–757. 18085475 10.1080/15216540701736285

[7] L. Schaller and V. M. Lauschke , “The Genetic Landscape of the Human Solute Carrier (SLC) Transporter Superfamily,” Human Genetics 138, no. 11–12 (2019): 1359–1377. 31679053 10.1007/s00439-019-02081-x PMC6874521

[8] A. Obaidat , M. Roth , and B. Hagenbuch , “The Expression and Function of Organic Anion Transporting Polypeptides in Normal Tissues and in Cancer,” Annual Review of Pharmacology and Toxicology 52 (2012): 135–151. 10.1146/annurev-pharmtox-010510-100556 PMC3257355 21854228

[9] M. Roth , A. Obaidat , and B. Hagenbuch , “OATPs, OATs and OCTs: The Organic Anion and Cation Transporters of the SLCO and SLC22A Gene Superfamilies,” British Journal of Pharmacology 165, no. 5 (2012): 1260–1287. 22013971 10.1111/j.1476-5381.2011.01724.x PMC3372714

[10] C. Colas , P. M. Ung , and A. Schlessinger , “SLC Transporters: Structure, Function, and Drug Discovery,” Medicinal Chemistry Communications 7, no. 6 (2016): 1069–1081. 27672436 10.1039/C6MD00005C PMC5034948

[11] R. Sutherland , A. Meeson , and S. Lowes , “Solute Transporters and Malignancy: Establishing the Role of Uptake Transporters in Breast Cancer and Breast Cancer Metastasis,” Cancer Metastasis Reviews 39, no. 3 (2020): 919–932. 32388639 10.1007/s10555-020-09879-6 PMC7497311

[12] N. Anzai , Y. Kanai , and H. Endou , “Organic Anion Transporter Family: Current Knowledge,” Journal of Pharmacological Sciences 100, no. 5 (2006): 411–426. 16799257 10.1254/jphs.crj06006x

[13] T. Nakanishi and I. Tamai , “Solute Carrier Transporters as Targets for Drug Delivery and Pharmacological Intervention for Chemotherapy,” Journal of Pharmaceutical Sciences 100, no. 9 (2011): 3731–3750. 21630275 10.1002/jps.22576

[14] B. Hagenbuch , “Cellular Entry of Thyroid Hormones by Organic Anion Transporting Polypeptides,” Best Practice & Research. Clinical Endocrinology & Metabolism 21, no. 2 (2007): 209–221. 17574004 10.1016/j.beem.2007.03.004

[15] B. Stieger and B. Hagenbuch , “Organic Anion‐Transporting Polypeptides,” Current Topics in Membranes 73 (2014): 205–232. 24745984 10.1016/B978-0-12-800223-0.00005-0 PMC3996503

[16] N. Thakkar , A. C. Lockhart , and W. Lee , “Role of Organic Anion‐Transporting Polypeptides (OATPs) in Cancer Therapy,” AAPS Journal 17, no. 3 (2015): 535–545. 25735612 10.1208/s12248-015-9740-x PMC4406968

[17] A. D. Ciută , K. Nosol , J. Kowal , et al., “Structure of Human Drug Transporters OATP1B1 and OATP1B3,” Nature Communications 14, no. 1 (2023): 5774. 10.1038/s41467-023-41552-8 PMC10507018 37723174

[18] N. Banerjee , C. Allen , and R. Bendayan , “Differential Role of Organic Anion‐Transporting Polypeptides in Estrone‐3‐Sulphate Uptake by Breast Epithelial Cells and Breast Cancer Cells,” Journal of Pharmacology and Experimental Therapeutics 342, no. 2 (2012): 510–519. 22588260 10.1124/jpet.112.192344

[19] A. Dodson , S. Parry , M. Ibrahim , et al., “Breast Cancer Biomarkers in Clinical Testing: Analysis of a UK National External Quality Assessment Scheme for Immunocytochemistry and in Situ Hybridisation Database Containing Results From 199 300 Patients,” Journal of Pathology: Clinical Research 4, no. 4 (2018): 262–273. 30066480 10.1002/cjp2.112 PMC6174620

[20] X. Dai , L. Xiang , T. Li , and Z. Bai , “Cancer Hallmarks, Biomarkers and Breast Cancer Molecular Subtypes,” Journal of Cancer 7, no. 10 (2016): 1281–1294. 27390604 10.7150/jca.13141 PMC4934037

[21] M. K. Tasoulis , J. Heil , and H. M. Kuerer , “De‐Escalating Surgery Among Patients With HER2 + and Triple Negative Breast Cancer,” Current Breast Cancer Reports 14, no. 4 (2022): 135–141. 35915668 10.1007/s12609-022-00453-3 PMC9328618

[22] C. Volk , “OCTs, OATs, and OCTNs: Structure and Function of the Polyspecific Organic Ion Transporters of the SLC22 Family,” Wiley Interdisciplinary Reviews: Membrane Transport and Signaling 3, no. 1 (2014): 1–13.

[23] A. T. Nies , H. Koepsell , K. Damme , and M. Schwab , “Organic Cation Transporters (OCTs, MATEs), In Vitro and In Vivo Evidence for the Importance in Drug Therapy,” Handbook of Experimental Pharmacology 201 (2011): 105–167. 10.1007/978-3-642-14541-4_3 21103969

[24] S. K. Nigam , “The SLC22 Transporter Family: A Paradigm for the Impact of Drug Transporters on Metabolic Pathways, Signaling, and Disease,” Annual Review of Pharmacology and Toxicology 58 (2018): 663–687. 10.1146/annurev-pharmtox-010617-052713 PMC6225997 29309257

[25] S. Douglass , A. P. Meeson , D. Overbeck‐Zubrzycka , et al., “Breast Cancer Metastasis: Demonstration That FOXP3 Regulates CXCR4 Expression and the Response to CXCL12,” Journal of Pathology 234, no. 1 (2014): 74–85. 24870556 10.1002/path.4381

[26] K. Mahkamova , N. Latar , S. Aspinall , and A. Meeson , “Hypoxia Increases Thyroid Cancer Stem Cell‐Enriched Side Population,” World Journal of Surgery 42, no. 2 (2018): 350–357. 29167950 10.1007/s00268-017-4331-x PMC5762807

[27] K. M. Britton , R. Eyre , I. J. Harvey , et al., “Breast Cancer, Side Population Cells and ABCG2 Expression,” Cancer Letters 323, no. 1 (2012): 97–105. 22521545 10.1016/j.canlet.2012.03.041 PMC3880937

[28] K. Mahkamova , N. Latar , S. Aspinall , and A. Meeson , “Side Population Cells in Anaplastic Thyroid Cancer and Normal Thyroid,” Experimental Cell Research 374 (2019): 104–113. 30465733 10.1016/j.yexcr.2018.11.012

[29] B. Isidor , O. Pichon , R. Redon , et al., “Mesomelia‐Synostoses Syndrome Results From Deletion of SULF1 and SLCO5A1 Genes at 8q13,” American Journal of Human Genetics 87, no. 1 (2010): 95–100. 20602915 10.1016/j.ajhg.2010.05.012 PMC2896765

[30] T. E. Wiese , L. G. Kral , K. E. Dennis , W. B. Butler , and S. C. Brooks , “Optimization of Estrogen Growth Response in MCF‐7 Cells,” In Vitro Cellular & Developmental Biology 28a, no. 9–10 (1992): 595–602. 1429362 10.1007/BF02631033

[31] J. M. Tian , B. Ran , C. L. Zhang , D. M. Yan , and X. H. Li , “Estrogen and Progesterone Promote Breast Cancer Cell Proliferation by Inducing Cyclin G1 Expression,” Brazilian Journal of Medical and Biological Research 51, no. 3 (2018): 1–7. 10.1590/1414-431X20175612 PMC5912097 29513878

[32] K. Wlcek , M. Svoboda , T. Thalhammer , F. Sellner , G. Krupitza , and W. Jaeger , “Altered Expression of Organic Anion Transporter Polypeptide (OATP) Genes in Human Breast Carcinoma,” Cancer Biology & Therapy 7, no. 9 (2008): 1450–1455. 18948755 10.4161/cbt.7.9.6282

[33] N. F. Smith , W. D. Figg , and A. Sparreboom , “Role of the Liver‐Specific Transporters OATP1B1 and OATP1B3 in Governing Drug Elimination,” Expert Opinion on Drug Metabolism & Toxicology 1, no. 3 (2005): 429–445. 16863454 10.1517/17425255.1.3.429

[34] T. Tang , G. Wang , S. Liu , et al., “Highly Expressed SLCO1B3 Inhibits the Occurrence and Development of Breast Cancer and Can Be Used as a Clinical Indicator of Prognosis,” Scientific Reports 11, no. 1 (2021): 631. 33436824 10.1038/s41598-020-80152-0 PMC7803962

[35] M. Muto , T. Onogawa , T. Suzuki , et al., “Human Liver‐Specific Organic Anion Transporter‐2 Is a Potent Prognostic Factor for Human Breast Carcinoma,” Cancer Science 98, no. 10 (2007): 1570–1576. 17760952 10.1111/j.1349-7006.2007.00570.x PMC11159603

[36] M. Okabe , G. Szakács , M. A. Reimers , et al., “Profiling SLCO and SLC22 Genes in the NCI‐60 Cancer Cell Lines to Identify Drug Uptake Transporters,” Molecular Cancer Therapeutics 7, no. 9 (2008): 3081–3091. 18790787 10.1158/1535-7163.MCT-08-0539 PMC2597359

[37] D. Iusuf , J. J. Hendrikx , A. van Esch , et al., “Human OATP1B1, OATP1B3 and OATP1A2 Can Mediate the In Vivo Uptake and Clearance of Docetaxel,” International Journal of Cancer 136, no. 1 (2015): 225–233. 24825069 10.1002/ijc.28970

[38] E. van de Steeg , A. van Esch , E. Wagenaar , K. E. Kenworthy , and A. H. Schinkel , “Influence of Human OATP1B1, OATP1B3, and OATP1A2 on the Pharmacokinetics of Methotrexate and Paclitaxel in Humanized Transgenic Mice,” Clinical Cancer Research 19, no. 4 (2013): 821–832. 23243220 10.1158/1078-0432.CCR-12-2080

[39] J. König , A. Seithel , U. Gradhand , and M. F. Fromm , “Pharmacogenomics of Human OATP Transporters,” Naunyn‐Schmiedeberg's Archives of Pharmacology 372, no. 6 (2006): 432–443. 16525793 10.1007/s00210-006-0040-y

[40] T. Nakanishi , Y. Nakamura , and J. Umeno , “Recent Advances in Studies of SLCO2A1 as a Key Regulator of the Delivery of Prostaglandins to Their Sites of Action,” Pharmacology & Therapeutics 223 (2021): 107803. 33465398 10.1016/j.pharmthera.2021.107803

[41] B. Hagenbuch and B. Stieger , “The SLCO (Former SLC21) Superfamily of Transporters,” Molecular Aspects of Medicine 34, no. 2–3 (2013): 396–412. 23506880 10.1016/j.mam.2012.10.009 PMC3602805

[42] S. J. McFeely , L. Wu , T. K. Ritchie , and J. Unadkat , “Organic Anion Transporting Polypeptide 2B1 – More Than a Glass‐Full of Drug Interactions,” Pharmacology & Therapeutics 196 (2019): 204–215. 30557631 10.1016/j.pharmthera.2018.12.009

[43] E. Rumiato , A. Brunello , S. Ahcene‐Djaballah , et al., “Predictive Markers in Elderly Patients With Estrogen Receptor‐Positive Breast Cancer Treated With Aromatase Inhibitors: An Array‐Based Pharmacogenetic Study,” Pharmacogenomics Journal 16, no. 6 (2016): 525–529. 26503812 10.1038/tpj.2015.73

[44] H. Yamaguchi , M. Sugie , M. Okada , et al., “Transport of Estrone 3‐Sulfate Mediated by Organic Anion Transporter OATP4C1: Estrone 3‐Sulfate Binds to the Different Recognition Site for Digoxin in OATP4C1,” Drug Metabolism and Pharmacokinetics 25, no. 3 (2010): 314–317. 20610891 10.2133/dmpk.25.314

[45] E. Taghikhani , R. Maas , M. F. Fromm , and J. König , “The Renal Transport Protein OATP4C1 Mediates Uptake of the Uremic Toxin Asymmetric Dimethylarginine (ADMA) and Efflux of Cardioprotective L‐Homoarginine,” PLoS One 14, no. 3 (2019): e0213747, 10.1371/journal.pone.0213747. 30865704 PMC6415861

[46] T. Mikkaichi , T. Suzuki , T. Onogawa , et al., “Isolation and Characterization of a Digoxin Transporter and Its Rat Homologue Expressed in the Kidney,” Proceedings of the National Academy of Sciences of the United States of America 101, no. 10 (2004): 3569–3574, 10.1073/pnas.0304987101. 14993604 PMC373503

[47] J. Kindla , T. T. Rau , R. Jung , et al., “Expression and Localization of the Uptake Transporters OATP2B1, OATP3A1 and OATP5A1 in Non‐Malignant and Malignant Breast Tissue,” Cancer Biology & Therapy 11, no. 6 (2011): 584–591. 21278488 10.4161/cbt.11.6.14533

[48] M. Baliu‐Piqué , A. Pandiella , and A. Ocana , “Breast Cancer Heterogeneity and Response to Novel Therapeutics,” Cancers 12, no. 11 (2020): 3271. 33167363 10.3390/cancers12113271 PMC7694303

[49] I. Y. Gong and R. B. Kim , “Impact of Genetic Variation in OATP Transporters to Drug Disposition and Response,” Drug Metabolism and Pharmacokinetics 28, no. 1 (2013): 4–18. 23047721 10.2133/dmpk.dmpk-12-rv-099

[50] J. M. Dempsey , K. M. Kidwell , C. L. Gersch , et al., “Effects of SLCO1B1 Polymorphisms on Plasma Estrogen Concentrations in Women With Breast Cancer Receiving Aromatase Inhibitors Exemestane and Letrozole,” Pharmacogenomics 20, no. 8 (2019): 571–580, 10.2217/pgs-2019-0020. 31190621 PMC6891932

[51] C. F. Hjorth , P. Damkier , T. B. Stage , et al., “Single‐Nucleotide Polymorphisms and the Effectiveness of Taxane‐Based Chemotherapy in Premenopausal Breast Cancer: A Population‐Based Cohort Study in Denmark,” Breast Cancer Research and Treatment 194, no. 2 (2022): 353–363, 10.1007/s10549-022-06596-2. 35501422 PMC9239972

[52] N. Thakkar , K. Kim , E. R. Jang , et al., “A Cancer‐Specific Variant of the SLCO1B3 Gene Encodes a Novel Human Organic Anion Transporting Polypeptide 1B3 (OATP1B3) Localized Mainly in the Cytoplasm of Colon and Pancreatic Cancer Cells,” Molecular Pharmaceutics 10, no. 1 (2013): 406–416. 23215050 10.1021/mp3005353

[53] M. Svoboda , F. Mungenast , A. Gleiss , et al., “Clinical Significance of Organic Anion Transporting Polypeptide Gene Expression in High‐Grade Serous Ovarian Cancer,” Frontiers in Pharmacology 9 (2018): 842. 30131693 10.3389/fphar.2018.00842 PMC6090214

[54] K. Alam , T. Farasyn , K. Ding , and W. Yue , “Characterization of Liver‐ and Cancer‐Type‐Organic Anion Transporting Polypeptide (OATP) 1B3 Messenger RNA Expression in Normal and Cancerous Human Tissues,” Drug Metabolism Letters 12, no. 1 (2018): 24–32. 29577869 10.2174/1872312812666180326110146 PMC6133766

