# Supplementary material for: Differential Expression Patterns of SLCO Solute Carriers in Human Breast Cancer Cell Lines and Tumour Samples
Source: J Cell Mol Med. 2025 Sep 3;29(17):e70838. doi: 10.1111/jcmm.70838 (PMC12408355; doi:10.1111/jcmm.70838)
Supplement: Supplementary file 1 — Tables S1–S3: jcmm70838‐sup‐0001‐TableS1‐S3.docx. [file JCMM-29-e70838-s001.docx]

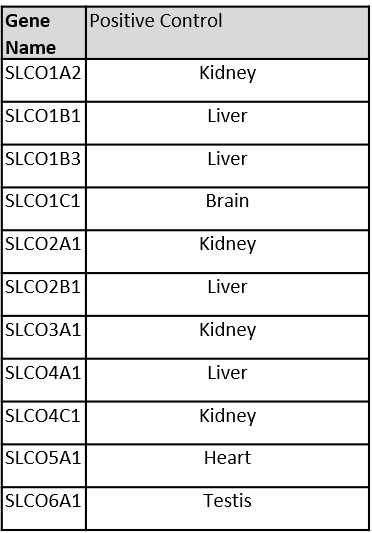


**Supp. Table 1.** Positive controls used for molecular analysis of SLCOs.

**Supp. Table 2: List of primer sequences, product lengths and Optimal PCR conditions.**


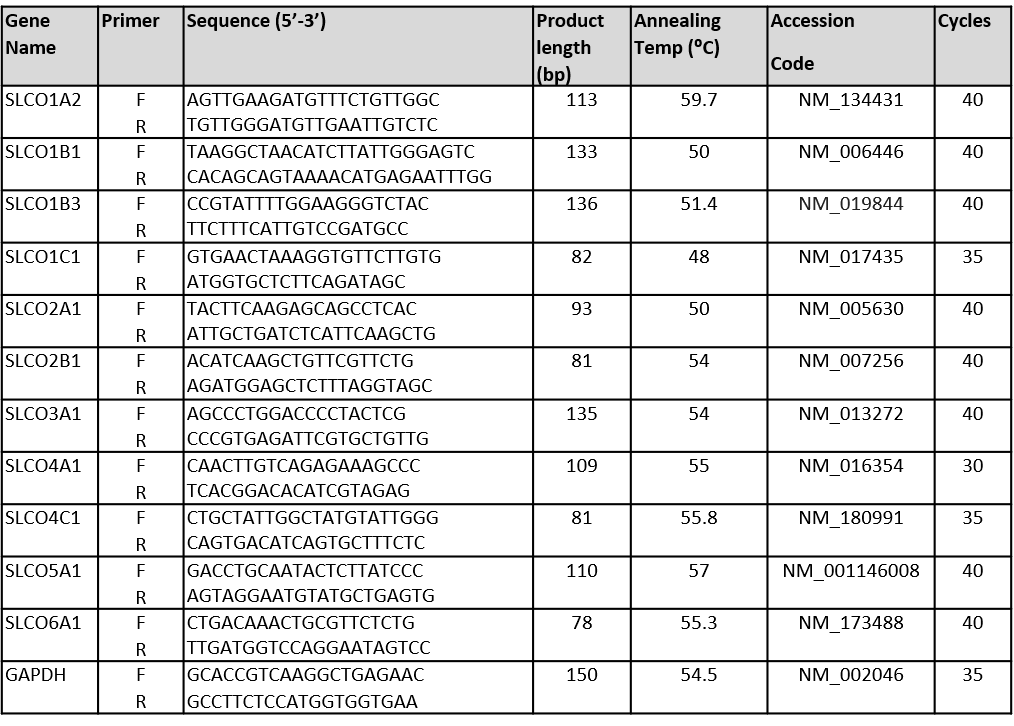


**Supp. Table 2:** List of primer sequences, product lengths and optimal PCR conditions

| **Target Name** | **Assay ID** |
| --- | --- |
| SLCO1A2 | Hs00366488_m1 |
| SLCO1B1 | Hs00272374_m1 |
| SLCO1B3 | Hs00251986_m1 |
| SLCO1C1 | Hs00213714_m1 |
| SLCO2A1 | Hs01114926_m1 |
| SLCO2B1 | Hs01030343_m1 |
| SLCO3A1 | Hs00203184_m1 |
| SLCO4A1 | Hs00249583_m1 |
| SLCO4C1 | Hs00698884_m1 |
| SLCO5A1 | Hs00229597_m1 |
| SLCO6A1 | Hs00542843_m1 |
| Beta Actin | Hs01060665_g1 |

**Supp. Table 3:** TaqMan gene expression assay targets and assay IDs.
